# Different Routes to Inhibit Fatty Acid Amide Hydrolase: Do All Roads Lead to the Same Place?

**DOI:** 10.3390/ijms20184503

**Published:** 2019-09-11

**Authors:** Giacomo Giacovazzo, Tiziana Bisogno, Fabiana Piscitelli, Roberta Verde, Sergio Oddi, Mauro Maccarrone, Roberto Coccurello

**Affiliations:** 1 Fondazione Santa Lucia IRCCS, Preclinical Neuroscience, Via del Fosso di Fiorano 64, 00143 Rome, Italy; 2 Endocannabinoid Research Group, Institute of Translational Pharmacology, CNR, Via Fosso del Cavaliere 100, 00133 Rome, Italy; 3 Endocannabinoid Research Group, Institute of Biomolecular Chemistry, CNR, Via C. Flegrei 34, 80078 Pozzuoli, Italy; 4 Faculty of Veterinary Medicine, University of Teramo, via R. Balzarini 1, 64100 Teramo, Italy; 5 Department of Medicine, Campus Bio-Medico University of Rome, Via Alvaro del Portillo 21, 00128 Rome, Italy; 6 Institute for Complex Systems (ISC), C.N.R., Via dei Taurini 19, 00185 Rome, Italy

**Keywords:** fatty acid amide hydrolase, intranasal drug delivery, URB597, PF-04457845, endocannabinoid tone, patients’ compliance

## Abstract

There is robust evidence indicating that enhancing the endocannabinoid (eCB) tone has therapeutic potential in several brain disorders. The inhibition of eCBs degradation by fatty acid amide hydrolase (FAAH) blockade, is the best-known option to increase *N*-acyl-ethanolamines-(NAEs)-mediated signaling. Here, we investigated the hypothesis that intranasal delivery is an effective route for different FAAH inhibitors, such as URB597 and PF-04457845. URB597 and PF-04457845 were subchronically administered in C57BL/6 male mice every other day for 20 days for overall 10 drug treatment, and compared for their ability to inhibit FAAH activity by the way of three different routes of administration: intranasal (i.n.), intraperitoneal (i.p.) and oral (p.o.). Lastly, we compared the efficacy of the three routes in terms of URB597-induced increase of NAEs levels in liver and in different brain areas. Results: We show that PF-04457845 potently inhibits FAAH regardless the route selected, and that URB597 was less effective in the brain after p.o. administration while reached similar effects by i.n. and i.p. routes. Intranasal URB597 delivery always increased NAEs levels in brain areas, whereas a parallel increase was not observed in the liver. By showing the efficacy of intranasal FAAH inhibition, we provide evidence that nose-to-brain delivery is a suitable alternative to enhance brain eCB tone for the treatment of neurodegenerative disorders and improve patients’ compliance.

## 1. Introduction 

Over twenty years of investigation of the endocannabinoid (eCB) system has led to the notion that a fine-tuning of eCB signaling may hold promising therapeutic potential in many clinically-relevant conditions, and that upregulation of eCB tone may not only restore pathologically decreased eCB levels, but also provide an autoprotective-like mechanism and contribute to slow disease development and minimize the unwanted effects due to the direct activation of cannabinoid type-1 (CB_1_) and type-2 (CB_2_) receptors [1,2,3,4,5]. A widely investigated possibility to increase eCB tone is by acting on the two major eCBs degrading enzymes, namely fatty acid amide hydrolase (FAAH) and monoacylglycerol lipase (MAGL). Indeed, FAAH and MAGL hydrolyze *N*-arachidonoyl-ethanolamine (AEA) [6,7] and 2-arachidonoyl-glycerol (2-AG) [8,9], the best characterized eCBs. 

Several inhibitors of AEA hydrolysis have shown their efficacy in a variety of pathological conditions and, specifically, in pain relief and control of nociceptive responses, osteoarthritis and neuropathic pain [10,11,12]. The efficacy of FAAH inhibitors has demonstrated also in gastrointestinal, neurological and psychiatric conditions, such as inflammatory bowel disease, multiple sclerosis, Parkinson’s disease, epilepsy, anxiety and depression [13,14,15,16,17]. Notably, FAAH inhibition also enhances the levels of other *N*-acyl-ethanolamines (NAEs) such as *N*-palmitoylethanolamine (PEA) and *N*-oleoylethanolamine (OEA), for which anti-inflammatory, analgesic, neuroprotective and anti-obesity effects have been reported [18,19,20]. The family of FAAH inhibitors is continuously increasing, as confirmed by the interest of several pharmaceutical companies (e.g., Eli Lilly, Pfizer, Bial-Portela & Ca. SA, Janssen Pharmaceutica) in their chemical synthesis and pharmacological characterization although some controversial results have been reported in clinical trials [21,22]. Among the plethora of FAAH inhibitors developed so far, the most part of preclinical studies has involved the use of URB597, an alkylcarbamic acid aryl esters-derived FAAH inhibitor [23]. Moreover, a recently developed benzylidine piperidine urea-derived compound, PF-04457845, has been shown to be safe when orally administered in osteoarthritis patients and in management of cannabis withdrawal syndrome [22,24]. Besides PF-04457845, also URB597 has been enlisted to be administered by the oral route in a clinical trial involving its therapeutic potential in schizophrenic patients (ClinicalTrials.gov Identifier: NCT00916201). On this ground, we asked whether the translational potential associated to the use of irreversible FAAH inhibitors such as URB597 and PF-04457845 (from now on URB and PF, respectively), could be evaluated at the light of different routes of administration. The parenteral (i.e., intraperitoneal (i.p.) way of administration represents the “route of choice” for almost all the reports on animal studies involving the effects of URB, being the remaining part confined to the practice of intragastric gavage (per os, p.o.). On the other hand, since its synthesis, PF has been characterized for being highly bioavailable in oral administration [25]. However, although the rationale for practicing the p.o. administration in mice relies in the necessity to successfully mimic the clinical context in which patients are asked to swallow their medication, there is a lack of knowledge about the effects of equivalent patient-oriented alternative routes of drug administration. From this view, other parenteral routes of administration such as intramuscular, intravascular and subcutaneous do not offer additional advantages for patients’ compliance as compared to the i.p. route. By contrast, the intranasal (i.n.) delivery of therapeutics appears comparable to the enteral oral administration in terms of patient-oriented practice and may represent an alternative option of treatment for many pathologies, principally for psychiatric and neurodegenerative diseases (NDs). There is a paucity of information concerning the effects of FAAH inhibitors via the different routes of administration, and specifically in comparison with the effects of i.n. delivery. Moreover, as far as we know, there are no data on the effect/efficacy of i.n. PF delivery, while a recent study focused on the effects on prosocial behavior reported i.n. administration of solid lipid nanoparticles-containing URB in rats [26]. 

Considering the amount of investigations exploiting the translational potential of FAAH inhibitors and the impact of i.n. drug administration, we investigated whether i.n. delivery was an effective route of administration for different chemical types of FAAH inhibitors (i.e., URB and PF). Hence, we assessed URB efficacy in terms of degree of FAAH inhibition and ability to increase NAE levels over three different routes of administration (i.p., p.o. and i.n.), and we also compared URB efficacy with the FAAH inhibition induced by PF through the same routes. 

## 2. Results

### 2.1. Effects of the Different Routes of Urb and Pf Administration on Faah Activity

**Figure 1:**
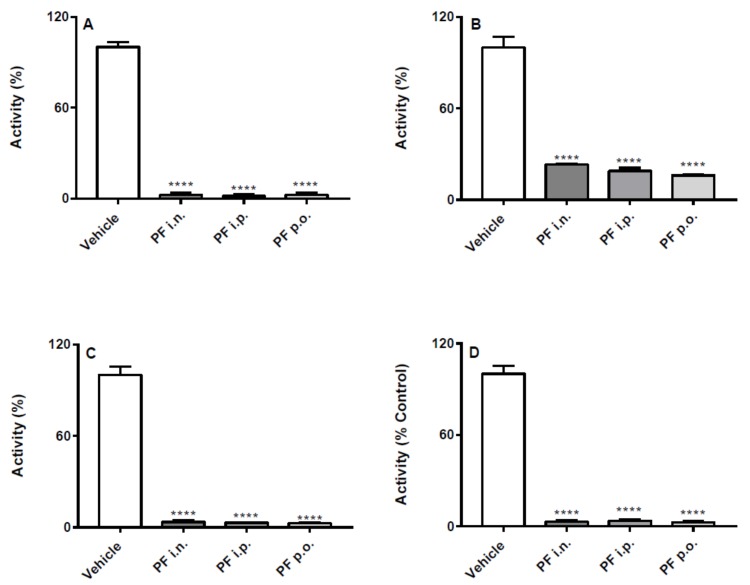
Effect of in vivo PF administration on FAAH activity. Inhibition of (**A**) hippocampus, (**B**) cortex, (**C**) cerebellum and (**D**) liver FAAH activity after the three different routes (i.n., i.p. or p.o.) of PF or vehicle administration. Results are means ± SEM expressed as percentage of the maximum effect observed in absence of PF plus vehicle administrated by the different routes. **** *p* < 0.0001 post hoc comparisons with vehicle conditions, Tukey’s test.

**Figure 2:**
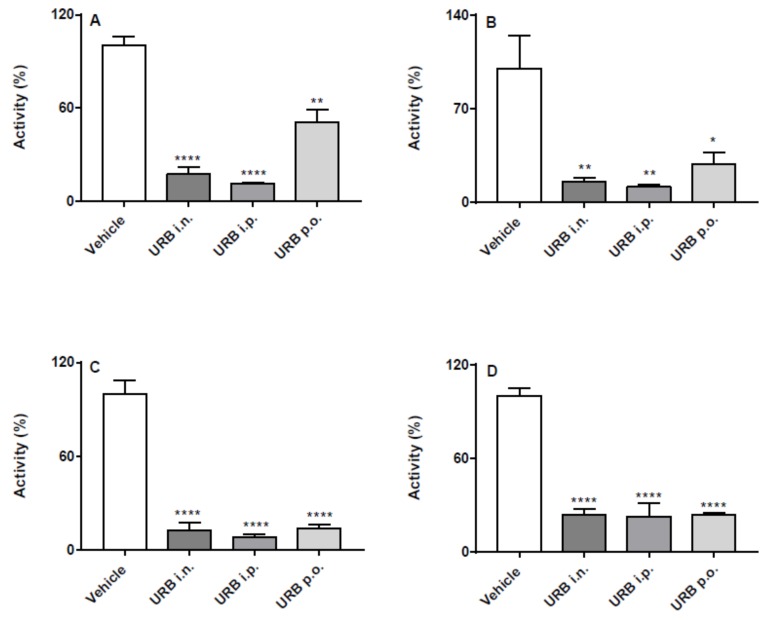
Effect of in vivo URB administration on FAAH activity. Inhibition of (**A**) hippocampus, (**B**) cortex, (**C**) cerebellum and (**D**) liver FAAH activity after the three different routes (i.n., i.p. or p.o.) of URB or vehicle administration. Results are means ± SEM expressed as percentage of the maximum effect observed in absence of URB plus vehicle administrated by the different routes. * *p* < 0.05, ** *p* < 0.01 and **** *p* < 0.0001 post hoc comparisons with vehicle conditions, Tukey’s test.

Both PF and URB significantly decreased FAAH activity in all brain areas and in liver, regardless of the route of administration (Figure 1 and Figure 2). In particular, none of the different routes significantly influenced neither quantitatively or qualitatively the degree of inhibition induced by PF administration (Figure 1). On the other hand, depending on the brain areas, i.p. and i.n. URB administration had a more pronounced effects with respect to p.o., whereas a comparable inhibitory effect was observed in the liver (Figure 2). 

### 2.2. Effect of the Different Routes of Urb Administration on Hippocampal Levels of AEA, PEA, OEA and 2-AG

**Figure 3:**
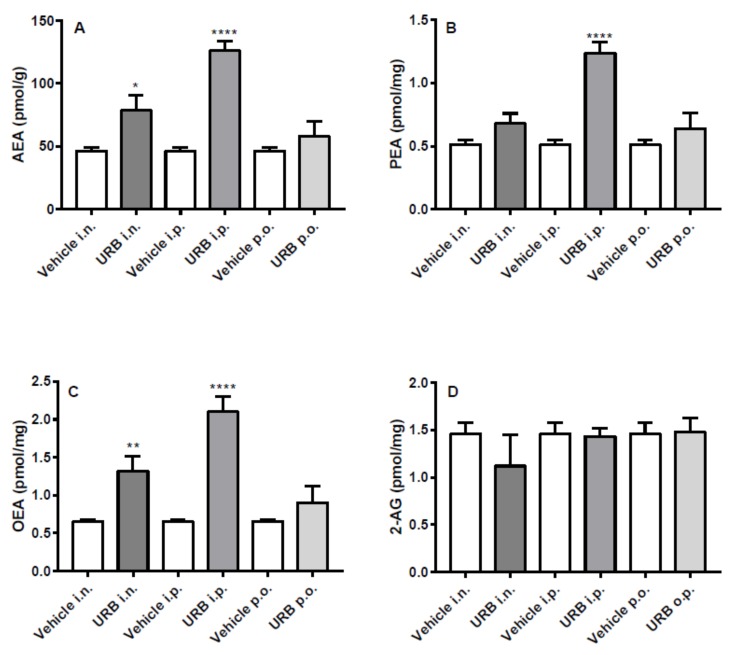
Effect of in vivo URB administration on hippocampal levels of NAEs and 2-AG. Levels of (**A**) AEA, (**B**) PEA, (**C**) OEA and (**D**) 2-AG following URB or vehicle (i.n., i.p. or p.o.) administration. Data are presented as mean ± SEM expressed as pmol per gram or milligram of wet tissue. * *p* < 0.05, ** *p* < 0.01 and **** *p* < 0.0001 post hoc comparisons with vehicle conditions, Tukey’s test.

The i.p. route of administration significantly increased AEA, PEA and OEA hippocampal levels in URB-treated mice. Moreover, the i.n. URB delivery caused an increase of NAE levels showing a profile very similar to that of i.p. administration, with the exception of PEA levels that did not reach statistical significance (Figure 3A–C). No significant differences were observed for AEA, PEA and OEA levels by p.o. URB administration, while none of the different routes of URB administration affected 2-AG levels (Figure 3D). 

### 2.3. Effect of the Different Routes of Urb Administration on Cortical Levels of AEA, PEA, OEA and 2-AG

**Figure 4:**
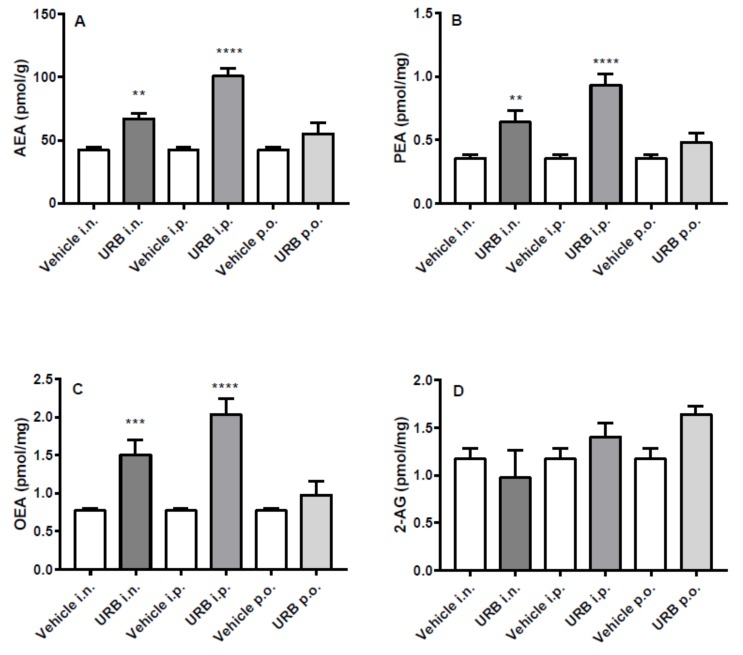
Effect of in vivo URB administration on cortical levels of NAEs and 2-AG. Levels of (**A**) AEA, (**B**) PEA, (**C**) OEA and (**D**) 2-AG following URB or vehicle (i.n., i.p. or p.o.) administration. Data are presented as mean ± SEM expressed as pmol per gram or milligram of wet tissue. ** *p* < 0.01, *** *p* < 0.001 and **** *p* < 0.0001 post hoc comparisons with vehicle conditions, Tukey’s test.

All three NAEs measured increased in the cortex of mice after i.p. or i.n. URB administration, while no differences were observed in p.o.-administered group (Figure 4A–C). Instead, the different routes of URB administration did not affect 2-AG levels (Figure 4D).

### 2.4. Effect of the Different Routes of Urb Administration on Cerebellum Levels of AEA, PEA, OEA and 2-AG

**Figure 5:**
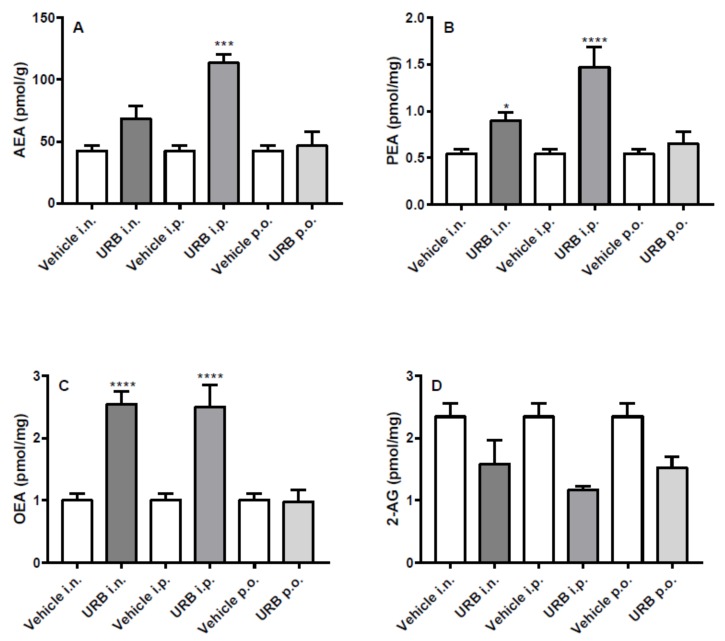
Effect of in vivo URB administration on cerebellum levels of NAEs and 2-AG. Levels of (**A**) AEA, (**B**) PEA, (**C**) OEA and (**D**) 2-AG following URB or vehicle (i.n., i.p. or p.o.) administration. Data are presented as mean ± SEM expressed as pmol per gram or milligram of wet tissue. * *p* < 0.01, *** *p* < 0.001 and **** *p* < 0.0001 post hoc comparisons with vehicle conditions, Tukey’s test.

In the cerebellum, all NAEs increased significantly after i.p. URB administration. Moreover, except for AEA that did not reach statistical significance, the other NAEs significantly increased after i.n. administration in a fashion similar to that observed by i.p. route. By contrast, p.o. URB administration failed to increase NAEs levels in the cerebellum and as the other routes of URB administration did not modify 2-AG levels (Figure 5).

### 2.5. Effect of the Different Routes of Urb Administration on Liver Levels of AEA, PEA, OEA and 2-AG

**Figure 6:**
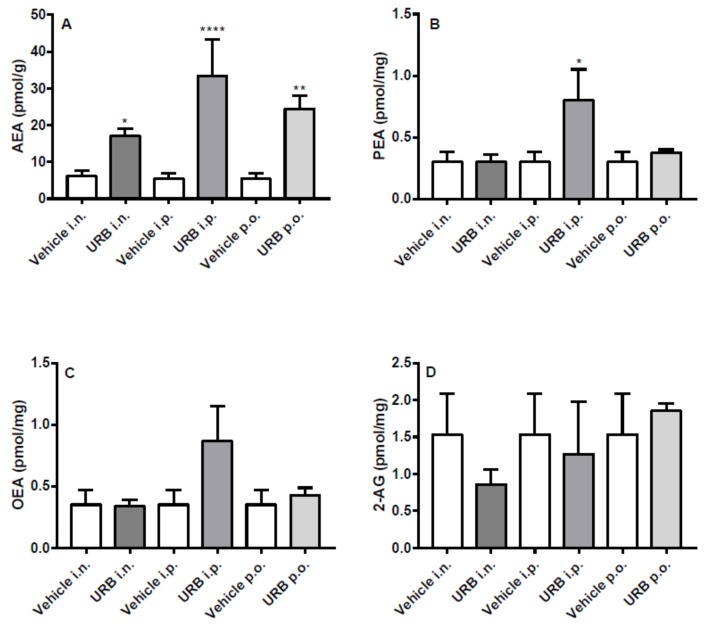
Effect of in vivo URB administration on liver levels of NAEs and 2-AG. Levels of (**A**) AEA, (**B**) PEA, (**C**) OEA and (**D**) 2-AG following URB or vehicle (i.n., i.p. or p.o.) administration. Data are presented as mean ± SEM expressed as pmol per gram or milligram of wet tissue. * *p* < 0.05, ** *p* < 0.01 and **** *p* < 0.0001 post hoc comparisons with vehicle conditions, Tukey’s test.

OEA and 2-AG levels in the liver were not affected by URB treatment, whatever the route of administration (Figure 6C–D). Except for the i.p. injection, PEA levels were never increased (Figure 6B). As for AEA levels in the liver, URB i.p. administration had the same effects as observed in the brain. On the other hand, URB p.o. administration that failed to increase NAEs levels within the brain, significantly enhanced AEA levels. Lastly, a slightly non-significant increase of AEA was caused by the i.n. route of administration (Figure 6A).

## 3. Discussion 

In the choice of a route of administration, safety, efficacy and easy accessibility should be considered as the most important principles for the selection of the best treatment option. Following these principles, the present data provide novel information about the effects observed in brain and in liver when in vivo FAAH inhibition is operated through three different routes of administration (i.p., i.n. and p.o.). Here, we assessed the FAAH inhibitory activity of the widely i.p. administered URB, and the orally-bioavailable PF in the different, never tested before, i.n. route of administration. Then, we compared the results obtained via the i.n. delivery with those from the conventionally used i.p. injection and p.o. administration. The dose administered for both inhibitors was selected for the lack of effects on motility, catalepsy and body temperature, as previously reported [27,28,29].

Our data are in agreement with FAAH inhibitory activity of PF via the p.o. route [25], as well as demonstrate that PF inhibits FAAH via the i.n. and i.p. administration. In this respect, PF shows similar central and peripheral efficacy regardless the route of administration selected. Most importantly, these findings suggest that the i.n. route holds comparable efficacy and potency, supporting i.n. as a viable way of delivery with major advantages in terms of patient compliance. On the other hand, the different routes of administration slightly affect URB-induced inhibition of FAAH. Indeed, while in the hepatic tissue URB efficacy through the i.n and p.o. routes were as strong as the i.p. administration, this was not detected in the brain. With the exception of cerebellum, we observe a reduced URB efficacy when the inhibitor was p.o. administered, while similar when i.n. and i.p. administered. Hence, the i.n. route resulted as effective as i.p., and may be therefore considered a suitable alternative also in the case of URB delivery. To validate our hypothesis, we investigated not only the effects of i.n. URB delivery on FAAH inhibition but also whether i.n. URB administration can increase liver and brain NAE levels, and whether the differences in the FAAH inhibition observed by the way of the three routes of URB administration are mirrored by the changes in the main FAAH substrates. Apart from a trend of increase of PEA in the hippocampus and AEA within the cerebellum, the i.n. URB delivery always increased NAEs in the three brain areas analyzed. Notably, the enhancement of NAE levels in the brain was not accompanied by the same increase of NAEs in the liver, despite the non-significant tendency towards an increase of AEA levels. Moreover, as previously reported for URB pharmacological profile [28,30], the oral administration of this inhibitor not only reduced FAAH activity but also increased NAE levels in peripheral tissues such as spinal cord. To the best of our knowledge, here we reported for the first time the lack of increase of NAE levels in several brain areas after oral URB administration, while we observed an increase of AEA at hepatic level. Conversely, as expected, i.p. URB administration increased NAE levels in the liver as well as in all brain areas analyzed. 

Together, these findings confirm the notion that i.n. drug administration is a promising route for the delivery of pharmacotherapeutics to the brain and, specifically, that enhancers of eCB signaling, such as FAAH inhibitors, are deliverable through the nose-to-brain pathway. We therefore demonstrate “on principle” that, in case of FAAH inhibition, the same drug formulation that was effective by i.p. administration can be also used for the i.n. route. From a translational point of view, and despite the i.p. route has allowed to achieve higher levels of NAEs increase, the effects of systemic i.p. administration are not comparable to those obtained via the i.n. delivery. Indeed, while the interest for the i.n. route is growing because its non-invasive impact and the simple and painless procedure for patients, other parenteral routes (e.g., intravenous) lack of the same requisites and cannot be self-administered. At difference with i.p. administration, i.n. URB delivery did not affect peripheral NAE levels, thus demonstrating that the use of FAAH inhibitors through the i.n. route can selectively increase brain NAE levels. Moreover, when the oral administration fails to deliver drug treatment to the brain or is associated to unwanted side effects, the nose-to-brain drug delivery is the only viable route of administration, especially in case of therapy of neurological or neuropsychiatric disorders. Several studies have been focusing the attention towards the mechanisms underlying nose-to-brain drug delivery [31,32], and the direct and indirect transport pathways including the systemic, olfactory and trigeminal nerve pathways [33,34]. The neuroepithelium, located at the roof of the nasal cavity, is a portion of the CNS not protected by the blood-brain barrier (BBB), which allow connecting the external environment to the brain via the olfactory and trigeminal nerve pathways. Although a combination of pathways is responsible for the nose-to-brain drug transport, one pathway may be favored over the others depending on the type of drug formulation, as well as on chemical features of the molecule delivered. Once intranasally delivered, the drug spreads to the internal wall of nasal cavity contacting both the blood vessels and the respiratory epithelium and the neural pathway including the olfactory epithelium [31,35]. Within the context of our data, we can estimate that i.n. URB delivery may have mostly targeted the brain through the indirect systemic pathway by absorption of URB from the vascular respiratory epithelium and then cross the BBB and the blood-cerebrospinal fluid barrier to the brain [33]. Indeed, although the mechanism of nose-to-brain transport is elusive for URB, and beyond our purpose, the lack of increase of NAEs content in the liver support the idea that both systemic and olfactory/trigeminal nerve pathways concurred to the delivery of URB to the brain. Recently either URB- or the CB1/CB2 receptor agonist HU-211-loaded solid lipid nanoparticles have been tested in vivo for social behavior or anti-depressant activity, respectively [26,36].

Since the aim of the study was to compare the effects of the same drug formulation in terms of FAAH inhibition and increase of the FAAH substrates in brain areas and at hepatic level, we did not test specific drug carriers for the nose-to-brain drug delivery. However, the present data do not rule out that the effects of i.n. delivery of FAAH inhibitors might be ameliorated by recently tested nanocarriers or chemical modifiers [26]. Thus, our data provide a direct demonstration of the efficacy of nose-to-brain delivery of FAAH inhibitors, highlighting that i.n. but not p.o. URB administration allows to achieve results matching those obtained via the parenteral i.p. URB administration. 

## 4. Materials and Methods

### 4.1. Animals, Dosage and Routes of Administration

Sixteen-weeks old male C57BL/6 mice were subchronically administered every other day for 20 days for a total of 10 drug treatment by the way of three different routes of administration, as follows: intraperitoneally (i.p.; URB N = 6, PF N = 4; Vehicle (VEH) N = 6), intranasally (i.n.; URB N = 6, PF N = 4; Vehicle (VEH) N = 6) and by oral gavage (p.o.; URB N = 6, PF N = 4; Vehicle (VEH) N = 6). The experiments were performed in accordance with Italian National Laws (DL 26/2014), with the European Communities Council Directive of September 22, 2010 (2010/63/EU) and the Guide for the Care and Use of Laboratory Animals (National Institutes of Health, Bethesda, MD, USA). The animals were kept on a 12:12 h light:dark cycle in a temperature- and humidity-controlled room (lights on at 0700 h), with water and standard laboratory chow provided *ad libitum*. 3-(3-carbamoylphenyl)phenyl *N*-cyclohexylcarbamate (URB) and *N*-3-p[yridazinyl-4-[[3-[[5-(trifluoromethyl)-2-pyridinyl]oxy]-phenyl]methylene]-1-piperidinecarboxamide (PF) both from Cayman Chemical Company (Ann Arbor, MI, USA) were dissolved in a fresh solution made of 10% polyethylene glycol (PEG) 400, 5% Tween 80 in sterile NaCl 0.9%. URB and PF were administered at 5 mg/kg for all the routes assessed. Oral and i.p. administration for both compounds were performed as previously reported [11]. URB dosage was chosen on the basis of previous studies showing the in vivo efficacy of this inhibitor in a dose range of 1 up to 10 mg/kg via i.p., p.o. or subcutaneous routes of administration [27,28,29]. Since orally effective at 1 mg/kg, non-catalepsigenic at 10 mg/kg, and reaching the maximum plasma concentration at 5 mg/kg [25], PF was administered at the same URB dosage. Concerning i.n. delivery, the URB volume has been determined accordingly to the constraints of the maximum volume compatible per each nostril with the procedure of i.n. drug infusion in mouse. All mice were sacrificed by cervical dislocation within 1 h after the last URB or PF administration, for all the routes of administration. Then, brain areas were dissected, liver removed, and all tissues stored at −80 °C until assays of FAAH activity and quantification of NAEs.

### 4.2. Assay of Fatty Acid Amide Hydrolase

The effect of the different routes of URB and PF administration on FAAH enzymatic activity was assessed, as previously reported [37]. In brief, tissues were homogenized at 4 °C in 50 mM Tris–HCl buffer, pH 7.0 and then were first centrifuged at 800× *g* to get rid of debris and the supernatant was centrifuged at 12,000× *g*. The pellets from this latter centrifugation were used for the assay. Membranes (70 μg) were incubated with [^14^C]AEA (2.4 μM, 5.0 mCi/mmol, Larodan AB, Solna, Sweden) as FAAH substrate in 50 mM Tris-HCl, pH = 9, for 30 min at 37 °C. [^14^C]Ethanolamine produced from [^14^C]AEA hydrolysis was measured, as previously reported [37], by scintillation counting of the aqueous phase after extraction of the incubation mixture with two volumes of CHCl_3_/CH_3_OH (1/1, *v*/*v*). FAAH enzymatic activity was reported as percentage of the maximum effect observed in absence of PF or URB plus vehicle administrated by the different routes.

### 4.3. Lipid Extraction and eCB Analysis

Brain areas and liver were dounce-homogenized and extracted with chloroform/methanol/Tris-HCl (50 mM, pH 7.4) (2:1:1, *v*/*v*) containing internal deuterated standards for PEA, OEA, AEA, and 2-AG quantification by isotope dilution (d4-PEA, d4-OEA, d8-AEA and d5-2-AG, Cayman Chemical). The lipid-containing organic phases were then purified by open bed chromatography on silica and aliquots of fractions containing NAEs and monoacylglycerols were analyzed by isotope dilution liquid chromatography/mass spectrometry (LC-MS). MS detection was performed by using values of *m/z* 304 and 300 (molecular ions + 1 for d4-PEA and PEA), *m/z* 330 and 326 (molecular ions + 1 for d4-OEA and OEA), *m/z* 356 and 348 (molecular ions + 1 for d8-AEA and AEA), and 384 and 379 (molecular ion +1 for d5-2-AG and 2-AG). The levels of eCBs were then calculated on the basis of their area ratios with the internal deuterated standard signal areas and their amounts expressed as picomoles were then normalized per gram or milligram of wet tissue.

### 4.4. Statistical Analysis

Results are expressed as the mean ± S.E.M. Data analysis was performed using GraphPad Prism software (GraphPad Software, San Diego, CA, USA). The significance of differences between groups was determined by one-way analysis of variance followed by Tukey’s test for multiple comparisons. A value of *p* < 0.05 was considered significant.

## 5. Conclusions

The nose-to-brain drug delivery may be considered a safe, effective and easy accessible route for the administration of FAAH inhibitors. The use of the intranasal route for the delivery of FAAH blockers provides an inhibitory profile comparable to that observed with parental drug delivery, thus representing a promising alternative for the selection of targeted drug delivery.

## Abbreviations

eCB	Endocannabinoid
FAAH	fatty acid amide hydrolase
i.n.	intranasal
i.p.	intraperitoneal
NAEs	*N*-acyl-ethanolamines
p.o.	oral

## References

[1] Cravatt B.F., Lichtman A.H. (2003). Fatty acid amide hydrolase: An emerging therapeutic target in the endocannabinoid system. Curr. Opin. Chem. Biol..

[2] Pertwee R.G. (2014). Elevating endocannabinoid levels: Pharmacological strategies and potential therapeutic applications. Proc. Nutr. Soc..

[3] Maccarrone M., Bab I., Bíró T., Cabral G.A., Dey S.K., Di Marzo V., Konje J.C., Kunos G., Mechoulam R., Pacher P. (2015). Endocannabinoid signaling at the periphery: 50 years after THC. Trends Pharmacol. Sci..

[4] Friedman D., French J.A., Maccarrone M. (2019). Safety, efficacy, and mechanisms of action of cannabinoids in neurological disorders. Lancet Neurol..

[5] Di Marzo V. (2008). Targeting the endocannabinoid system: To enhance or reduce?. Nat. Rev. Drug Discov..

[6] Cravatt B.F., Giang D.K., Mayfield S.P., Boger D.L., Lerner R.A., Gilula N.B. (1996). Molecular characterization of an enzyme that degrades neuromodulatory fatty-acid amides. Nature.

[7] Maccarrone M. (2017). Metabolism of the Endocannabinoid Anandamide: Open Questions after 25 Years. Front. Mol. Neurosci..

[8] Karlsson M., Contreras J.A., Hellman U., Tornqvist H., Holm C. (1997). cDNA Cloning, Tissue Distribution, and Identification of the Catalytic Triad of Monoglyceride Lipase. J. Biol. Chem..

[9] Baggelaar M.P., Maccarrone M., van der Stelt M. (2018). 2-Arachidonoylglycerol: A signaling lipid with manifold actions in the brain. Prog. Lipid Res..

[10] Ahn K., Johnson D.S., Mileni M., Beidler D., Long J.Z., McKinney M.K., Weerapana E., Sadagopan N., Liimatta M., Smith S.E. (2009). Discovery and Characterization of a Highly Selective FAAH Inhibitor that Reduces Inflammatory Pain. Chem. Biol..

[11] Caprioli A., Coccurello R., Rapino C., Di Serio S., Di Tommaso M., Vertechy M., Vacca V., Battista N., Pavone F., Maccarrone M. (2012). The novel reversible fatty acid amide hydrolase inhibitor ST4070 increases endocannabinoid brain levels and counteracts neuropathic pain in different animal models. J. Pharmacol. Exp. Ther..

[12] Starowicz K., Di Marzo V. (2013). Non-psychotropic analgesic drugs from the endocannabinoid system: “Magic bullet” or “multiple-target” strategies?. Eur. J. Pharmacol..

[13] Baker D., Pryce G., Croxford J.L., Brown P., Pertwee R.G., Makriyannis A., Khanolkar A., Layward L., Fezza F., Bisogno T. (2001). Endocannabinoids control spasticity in a multiple sclerosis model. FASEB J..

[14] Micale V., Di Marzo V., Sulcova A., Wotjak C.T., Drago F. (2013). Endocannabinoid system and mood disorders: Priming a target for new therapies. Pharmacol. Ther..

[15] Vilela L.R., Medeiros D.C., Rezende G.H.S., de Oliveira A.C.P., Moraes M.F.D., Moreira F.A. (2013). Effects of cannabinoids and endocannabinoid hydrolysis inhibition on pentylenetetrazole-induced seizure and electroencephalographic activity in rats. Epilepsy Res..

[16] Hasenoehrl C., Storr M., Schicho R. (2017). Cannabinoids for treating inflammatory bowel diseases: Where are we and where do we go?. Expert Rev. Gastroenterol. Hepatol..

[17] Chiurchiù V., van der Stelt M., Centonze D., Maccarrone M. (2018). The endocannabinoid system and its therapeutic exploitation in multiple sclerosis: Clues for other neuroinflammatory diseases. Prog. Neurobiol..

[18] Provensi G., Coccurello R., Umehara H., Munari L., Giacovazzo G., Galeotti N., Nosi D., Gaetani S., Romano A., Moles A. (2014). Satiety factor oleoylethanolamide recruits the brain histaminergic system to inhibit food intake. Proc. Natl. Acad. Sci..

[19] Iannotti F.A., Di Marzo V., Petrosino S. (2016). Endocannabinoids and endocannabinoid-related mediators: Targets, metabolism and role in neurological disorders. Prog. Lipid Res..

[20] Passani M.B., Coccurello R. (2016). The Endocannabinoid-Like Derivative Oleoylethanolamide at the Gut–Brain Interface: A “Lipid Way” to Control Energy Intake and Body Weight. Cannabinoids in Health and Dis..

[21] Van Esbroeck A.C.M., Janssen A.P.A., Cognetta A.B., Ogasawara D., Shpak G., Van Der Kroeg M., Kantae V., Baggelaar M.P., De Vrij F.M.S., Deng H. (2017). Activity-based protein profiling reveals off-target proteins of the FAAH inhibitor BIA. Science.

[22] D’Souza D.C., Cortes-Briones J., Creatura G., Bluez G., Thurnauer H., Deaso E., Bielen K., Surti T., Radhakrishnan R., Gupta A. (2019). Efficacy and safety of a fatty acid amide hydrolase inhibitor (PF-04457845) in the treatment of cannabis withdrawal and dependence in men: A double-blind, placebo-controlled, parallel group, phase 2a single-site randomised controlled trial. Lancet Psychiatry.

[23] Mor M., Rivara S., Lodola A., Plazzi P.V., Tarzia G., Duranti A., Tontini A., Piersanti G., Kathuria S., Piomelli D. (2004). Cyclohexylcarbamic acid 3′- or 4′-substituted biphenyl-3-yl esters as fatty acid amide hydrolase inhibitors: Synthesis, quantitative structure-activity relationships, and molecular modeling studies. J. Med. Chem..

[24] Huggins J.P., Smart T.S., Langman S., Taylor L., Young T. (2012). An efficient randomised, placebo-controlled clinical trial with the irreversible fatty acid amide hydrolase-1 inhibitor PF-04457845, which modulates endocannabinoids but fails to induce effective analgesia in patients with pain due to osteoarthritis of the knee. Pain.

[25] Johnson D.S., Stiff C., Lazerwith S.E., Kesten S.R., Fay L.K., Morris M., Beidler D., Liimatta M.B., Smith S.E., Dudley D.T. (2011). Discovery of PF-04457845: A highly potent, orally bioavailable, and selective urea FAAH inhibitor. ACS Med. Chem. Lett..

[26] Esposito E., Drechsler M., Mariani P., Carducci F., Servadio M., Melancia F., Ratano P., Campolongo P., Trezza V., Cortesi R. (2017). Lipid nanoparticles for administration of poorly water soluble neuroactive drugs. Biomed. Microdevices.

[27] Maione S., De Petrocellis L., De Novellis V., Moriello A.S., Petrosino S., Palazzo E., Rossi F.S., Woodward D.F., Di Marzo V. (2007). Analgesic actions of N-arachidonoyl-serotonin, a fatty acid amide hydrolase inhibitor with antagonistic activity at vanilloid TRPV1 receptors. Br. J. Pharmacol..

[28] Russo R., LoVerme J., La Rana G., Compton T.R., Parrott J., Duranti A., Tontini A., Mor M., Tarzia G., Calignano A. (2007). The Fatty Acid Amide Hydrolase Inhibitor URB597 (Cyclohexylcarbamic Acid 3′-Carbamoylbiphenyl-3-yl Ester) Reduces Neuropathic Pain after Oral Administration in Mice. J. Pharmacol. Exp. Ther..

[29] Kwilasz A.J., Abdullah R.A., Poklis J.L., Lichtman A.H., Negus S.S. (2014). Effects of the fatty acid amide hydrolase inhibitor URB597 on pain-stimulated and pain-depressed behavior in rats. Behav. Pharmacol..

[30] Piomelli D., Tarzia G., Duranti A., Tontini A., Mor M., Compton T.R., Dasse O., Monaghan E.P., Parrott J.A., Putman D. (2006). Pharmacological profile of the selective FAAH inhibitor KDS-4103 (URB597). CNS Drug Rev..

[31] Crowe T.P., Greenlee M.H.W., Kanthasamy A.G., Hsu W.H. (2018). Mechanism of intranasal drug delivery directly to the brain. Life Sci..

[32] Dong X. (2018). Current strategies for brain drug delivery. Theranostics.

[33] Kozlovskaya L., Abou-Kaoud M., Stepensky D. (2014). Quantitative analysis of drug delivery to the brain via nasal route. J. Control. Release.

[34] Mittal D., Ali A., Md S., Baboota S., Sahni J.K., Ali J. (2014). Insights into direct nose to brain delivery: Current status and future perspective. Drug Deliv..

[35] Dhuria S.V., Hanson L.R., Frey W.H. (2010). Intranasal delivery to the central nervous system: Mechanisms and experimental considerations. J. Pharm. Sci..

[36] He X., Zhu Y., Wang M., Jing G., Zhu R., Wang S. (2016). Antidepressant effects of curcumin and HU-211 coencapsulated solid lipid nanoparticles against corticosterone-induced cellular and animal models of major depression. Int. J. Nanomedicine.

[37] Cascio M.G., Minassi A., Ligresti A., Appendino G., Burstein S., Di Marzo V. (2004). A structure-activity relationship study on N -arachidonoyl-amino acids as possible endogenous inhibitors of fatty acid amide hydrolase. Biochem. Biophys. Res. Commun..

